# Prognostic implication of time in target range of systolic blood pressure in patients with hypertension after coronary artery bypass grafting

**DOI:** 10.3389/fcvm.2026.1869459

**Published:** 2026-09-21

**Authors:** Yue Song, Qian Li, Qi Huang

**Affiliations:** Department of Cardiac Surgery, Beijing Anzhen Hospital, Capital Medical University, Beijing, China

**Keywords:** coronary artery bypass grafting, hypertension, medication adherence, systolic blood pressure, time in target range

## Abstract

**Objective:**

To evaluate the prognostic value of systolic blood pressure (SBP) time in target range (TTR) in patients with hypertension following coronary artery bypass grafting (CABG).

**Methods:**

We performed a retrospective analysis on patients with hypertension who underwent CABG between January 2022 and May 2024 A total of 528 patients with hypertension after CABG were included in this retrospective analysis. SBP target range was deﬁned as <140 mmHg. The outcome was the occurrence within 1 year of a major adverse cardiac or cerebrovascular event, defined as death from any cause, myocardial infarction, stroke, or repeat revascularization. We used Cox proportional hazards regression models to assess the association of SBP-TTR and clinical outcome.

**Results:**

A total of 528 patients with hypertension after CABG were included in this *post hoc* analysis. After multivariable adjustment, a 10% increase in SBP-TTR was associated with 22% decreased risk of adverse events [hazard ratio, 0.78 (95% CI, 0.67–0.90); *P* = 0.001]. Compared to those with a lower SBP-TTR, patients with SBP-TTR > 75%–100% were characterized by younger age, lower baseline BP, superior rate of DBP goal attainment and medication adherence. Kaplan–Meier curves demonstrated that higher SBP-TTR was associated with a significantly lower event probability over time (Log-rank *P* < 0.001).

**Conclusions:**

For hypertensive patients undergoing CABG, a higher SBP-TTR is associated with an improved clinical prognosis. Efforts to enhance medication adherence represent a promising strategy to increase target achievement.

## Introduction

Hypertension is a critical modifiable risk factor for adverse cardiovascular outcomes in patients following coronary artery surgery (1). While Coronary Artery Bypass Grafting (CABG) is the established treatment for complex coronary artery disease, postoperative patients remain at high risk for myocardial infarction (MI), stroke, and cardiovascular death (2, 3). The benefits and long-term outcomes of CABG may also vary according to patient characteristics, ventricular function, and operative strategy. In patients with ischemic cardiomyopathy, the survival benefit of CABG differed according to left ventricular end-systolic volume, age, and renal function, while right ventricular dysfunction was associated with a worse prognosis (4, 5). Although off-pump and on-pump CABG showed comparable long-term survival in selected patients, randomized evidence indicated a higher risk of graft occlusion with off-pump CABG, particularly for saphenous vein grafts and grafts to the left anterior descending and left circumflex territories (6, 7). These findings highlight the importance of individualized postoperative risk assessment and sustained secondary prevention, including effective systolic blood pressure (SBP) control (8).

Considerable debate persists regarding the optimal approach to blood pressure management (9). Traditional assessments relying on single clinical measurements fail to capture the dynamic BP fluctuations caused by surgical stress and fluid shifts. In contrast, time in target range (TTR) provides a comprehensive reflection of BP stability (10). Although higher TTR is associated with improved outcomes in atrial fibrillation and diabetic patients post-CABG, its specific relationship with prognosis in hypertensive CABG patients remains unclear (11, 12).

This study aims to investigate the association between SBP-TTR and the prognosis of hypertensive patients following CABG. It also explores a more optimal BP management target to prevent adverse events after revascularization.

## Methods

### Study design and population

This retrospective study analyzed data from the HeartMed Digital Management Center (Chengdu, China). We screened patients with hypertension who underwent CABG and completed at least 12 months of follow-up. Hypertension was defined as an SBP ≥ 140 mmHg and/or a DBP ≥ 90 mmHg, or current use of antihypertensive medication, according to the 2024 Chinese hypertension guideline (13). Patients without BP records or with fewer than five valid measurement days per month were excluded. The final cohort comprised 528 participants (Figure 1). The study was approved by the Beijing Anzhen Hospital IRB (No. KS2025139), with informed consent waived due to its retrospective nature.

**Figure 1:**
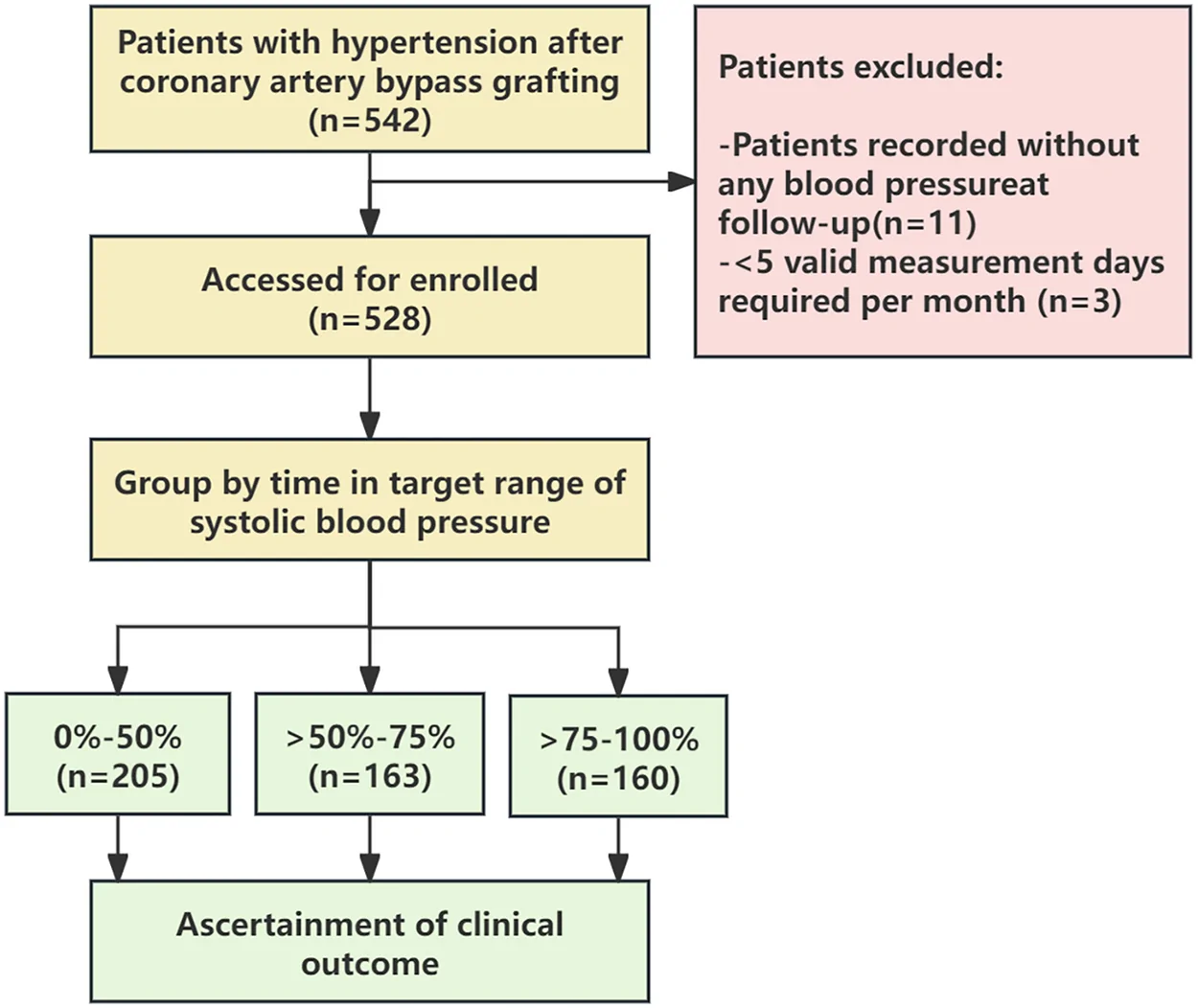
Flowchart of study participation.

### Data collection and outcome ascertainment

Clinical data—including demographics, comorbidities, discharge medications, and procedural characteristics—were extracted from the HeartMed database and cross-verified by two independent investigators. The primary clinical outcome was the occurrence of Major Adverse Cardiac and Cerebrovascular Events (MACCE), defined as all-cause death, MI, stroke, or repeat revascularization. Outcomes were adjudicated by study physicians through medical record reviews and telephone interviews.

### SBP-TTR calculation

Post-discharge blood pressure and resting heart rate were measured using a certified electronic sphygmomanometer (Bioland A223). Patients were instructed to remain seated quietly for at least 5 min before measuring BP at approximately 8:00 AM and 8:00 PM each day. Two readings were obtained at each session, and their mean was used as the session-level value. When both morning and evening measurements were available, their mean was used as the daily BP value; when only one session was available, that value was used. A valid measurement day was defined as a day with at least one valid session, and a valid follow-up month as a month with at least five valid measurement days.

The SBP threshold of 140 mmHg was prespecified according to the clinical BP management protocol applied during the study period and was anchored to the initial BP control target recommended by the 2024 Chinese hypertension guideline. SBP-TTR was defined as the proportion of evaluable follow-up time during which interpolated SBP remained below 140 mmHg. When the interpolated trajectory crossed the threshold, only the portion of the interval below 140 mmHg was counted (14, 15). Further details of the calculation algorithm are provided in the Supplementary Methods. Patients were categorized as having low (0%–50%), intermediate (>50%–75%), or high (>75%–100%) SBP-TTR.

Measurement completeness was assessed using the number of valid measurement days, the proportion of missing scheduled sessions, and within-patient variability in daily measurement frequency. The coefficient of variation was calculated as the standard deviation divided by the mean daily number of completed measurement sessions and expressed as a percentage.

### Statistical analyses

Analyses were conducted using R software (version 4.3.2). Baseline characteristics were compared across TTR groups using one-way ANOVA or Kruskal–Wallis tests for continuous variables (expressed as mean ± SD) and the chi-squared test for categorical variables. The association between SBP-TTR and clinical outcomes was evaluated using unadjusted and multivariable-adjusted Cox proportional hazards models to estimate hazard ratios (HRs) and 95% confidence intervals (CIs). Cumulative incidence was visualized using Kaplan–Meier curves and compared via the log-rank test. A two-sided *P* value < 0.05 was considered statistically significant.

Candidate covariates were selected according to their established clinical relevance to prognosis after CABG, evidence from previous studies, and associations with MACCEs in univariable Cox regression analyses. Variables with a univariable *P* value < 0.10 were considered for inclusion; however, univariable statistical significance was used only as a supportive criterion and not as the sole basis for selection. The primary multivariable model included SBP-TTR, age, BMI, mean SBP, heart rate time in target range, NYHA class III/IV, prior stroke, triglyceride level, and length of postoperative hospital stay. Given the limited number of outcome events, model complexity was restricted. A parsimonious sensitivity model including SBP-TTR, age, sex, BMI, mean SBP, and prior stroke was fitted to assess the robustness of the primary findings.

To assess the robustness of the findings to a more stringent BP target, SBP-TTR was also recalculated using an SBP threshold of 130 mmHg and analyzed as a continuous variable per 10% increase using the same multivariable Cox regression model and covariate set as in the primary analysis. To further evaluate the relationship between medication use and SBP-TTR, baseline use of the medication including *β*-blockers, ACEIs/ARBs, calcium channel blockers, statins, clopidogrel/ticagrelor, diuretics, and aspirin, was examined. All baseline medication variables were entered simultaneously into a multivariable linear regression model with SBP-TTR as the dependent variable after adjustment. To assess whether baseline medication use materially attenuated the association between SBP-TTR and MACCEs, each medication class was additionally entered, one at a time, into the primary multivariable Cox regression model. Medication classes were evaluated separately in the Cox models to limit model complexity given the number of observed outcome events.

## Results

### Baseline demographic and clinical characteristics

A total of 542 patients with hypertension who underwent CABG were derived from database between January 2022 and May 2024. After excluding patients without eligible SBP measurements (*n* = 14), 528 patients were included in this study finally, with 59 (11.2%) experiencing endpoint events during the one-year observation period. As shown in Supplementary Table S1, patients contributed a median of 298 valid measurement days (IQR, 272–326) during the 12-month follow-up. Overall, 20.2% of scheduled BP measurement sessions were missing. The median within-patient coefficient of variation in daily measurement frequency was 49.0% (IQR, 36.0%–61.2%). Table 1 shows the preoperative characteristics and procedural details of 528 patients. The mean age of the patients was 66 years, 118 (22.3%) were female. Patients experiencing events were elder, more likely had higher BMI, and higher prevalence of stroke. The mean SBP and SBP-TTR achieved during 12 months of study population were 128.7 ± 25.5 mm Hg and 57.9 ± 25.7%, respectively. Patients who experienced MACCEs had a higher mean SBP and a lower SBP-TTR than those who did not experience MACCEs.

**Table 1:** Baseline demographic, clinical, angiographic, and procedural characteristics according to clinical outcomes.

Characteristics	All patients (*n* = 528)	Event (+) (*n* = 59)	Event (−) (*n* = 469)	*P*-value
Demographic and clinical characteristics				
Age in yr	65.9 ± 12.6	71.1 ± 12.3	65.3 ± 12.5	**0** **.** **001**
Female, %	118 (22.3)	12 (20.3)	106 (22.6)	0.69
BMI, kg/m^2^	25.4 ± 4.8	26.9 ± 7.1	25.3 ± 4.3	**0** **.** **01**
Smoke, %	82 (15.5)	11 (18.6)	71 (15.1)	0.45
Drinking, %	43 (8.1)	6 (10.2)	37 (7.9)	0.46
Mean SBP, mmHg	128.7 ± 25.5	137.0 ± 22.4	127.7 ± 25.7	**0** **.** **008**
Mean DBP, mmHg	75.9 ± 10.7	75.8 ± 10.4	75.9 ± 10.8	0.97
Mean HR, bpm	68.9 ± 11.8	68.2 ± 13.8	69.0 ± 11.6	0.59
TTR-SBP, %	57.9 ± 25.7	42.2 ± 25.3	59.9 ± 25.1	**<0** **.** **001**
TTR-DBP, %	81.1 ± 30.1	76.4 ± 31.0	81.7 ± 29.9	0.20
TTR-heart rate, %	79.8 ± 32.6	71.2 ± 34.7	80.9 ± 32.3	**0** **.** **03**
NYHA class III/IV, %	72 (13.6)	10 (16.9)	62 (13.2)	0.59
Medical comorbidity, *n* (%)				
Stroke	43 (8.1)	9 (15.3)	34 (7.2)	**0** **.** **04**
Diabetes	184 (34.8)	26 (44.1)	158 (33.7)	0.25
Hyperlipidemia	51 (9.7)	7 (11.9)	44 (9.4)	0.70
Kidney disease	45 (8.5)	6 (10.2)	39 (8.3)	0.78
History of MI	71 (13.4)	9 (15.3)	62 (13.2)	0.85
Hyperuricemia	128 (24.2)	16 (27.1)	112 (23.9)	0.85
Laboratory measurements				
LVEF, %	58.5 ± 12.0	60.3 ± 7.4	58.3 ± 12.4	0.10
Ast, u/L	23.7 ± 14.6	25.4 ± 11.8	23.4 ± 14.9	0.34
Alt, u/L	25.5 ± 24.0	27.6 ± 19.3	25.3 ± 24.5	0.51
Creatinine, μmol/L	82.6 ± 48.9	79.4 ± 23.7	83.0 ± 51.1	0.62
LDL, mmol/L	2.3 ± 0.78	2.2 ± 0.66	2.3 ± 0.80	0.26
HDL, mmol/L	1.3 ± 0.43	1.3 ± 0.52	1.3 ± 0.42	0.63
TC, mmol/L	4.8 ± 1.0	4.6 ± 1.1	4.4 ± 1.0	0.19
TG, mmol/L	1.5 ± 0.84	1.7 ± 0.99	1.5 ± 0.81	0.09
Angiographic and procedural characteristics				
Procedure time, min	151.7 ± 37.2	158.5 ± 35.7	150.0 ± 37.3	0.14
Length of hospital stay, d	9.5 ± 2.6	10.3 ± 3.0	9.4 ± 2.5	**0** **.** **01**
No. of lesions	4.0 ± 1.2	4.3 ± 1.3	4.0 ± 1.2	0.11
Multiple arterial grafts, %	133 (25.2)	19 (32.2)	114 (24.3)	0.19
LIMA used as graft, %	521 (98.7)	58 (98.3)	463 (98.7)	0.57

Values are mean ± SD or *n* (%).Bold values indicate statistically significant differences (*P* < 0.05).

BMI, body mass index; TTR, time in target range; SBP, systolic blood pressure; DBP, diastolic blood pressure; NYHA, New York Heart Association; MI, myocardial infarction; LVEF, left ventricular ejection fraction; Ast, aspartate aminotransferase; Alt, alanine aminotransferase; LDL, low-density lipoprotein; HDL, high-density lipoprotein; TC, total cholesterol; TG, triglycerides and LIMA, left internal mammary artery.

### Potential predictors of composite endpoint events

Univariate Cox regression identified age (HR: 1.04, 95% CI: 1.02–1.07), BMI (HR: 1.05, 95% CI: 1.01–1.09), baseline SBP (HR: 1.01, 95% CI: 1.00–1.02), SBP-TTR (HR: 0.76, 95% CI: 0.68–0.84), HR TTR (HR: 0.48 95% CI: 0.24–0.94), previous stroke (HR: 2.32, 95% CI: 1.14–4.71) and length of hospital stay (HR: 1.14, 95% CI: 1.03–1.26) as significant factors associated with cumulative clinical events. Subsequent multivariate analysis, adjusted for confounders, demonstrated that age (HR: 1.04, 95% CI: 1.01–1.08), SBP-TTR (HR: 0.78, 95% CI: 0.67–0.90) and previous stroke (HR: 3.28, 95% CI: 1.49–7.21) remained independently associated with adverse outcomes in patients. Each 10% increase in SBP-TTR was signiﬁcantly associated with 22% decreased risk of adverse events (Table 2). In the sensitivity analysis using the more stringent SBP threshold of 130 mmHg, each 10% increase in SBP-TTR was associated with a 27% lower risk of MACCEs (HR: 0.73; 95% CI, 0.60–0.89; *P* = 0.002), consistent with the primary analysis using the 140 mmHg threshold (Supplementary Table S2). The association remained consistent in the parsimonious sensitivity model. Each 10% increase in SBP-TTR was associated with a 21% lower risk of MACCEs after adjustment for age, sex, BMI, mean SBP, and prior stroke (HR: 0.79; 95% CI, 0.70–0.89; *P* < 0.001; Supplementary Table S5).

**Table 2:** Cox regression analysis of single and multiple factors related to PO.

Observed items	Single factors			Multiple factors		
	HR	95%CI	*P*-value	HR	95%CI	*P*-value
Age	1.04	1.02–1.07	**<0** **.** **001**	1.04	1.01–1.08	0.01
Female	0.87	0.55–1.63	0.66	Not selected		
BMI	1.05	1.01–1.09	**0** **.** **01**	—		
Smoke	0.29	0.67–2.49	0.44	Not selected		
Drinking	1.31	0.56–3.05	0.53	Not selected		
Mean SBP	1.01	1.00–1.02	**0** **.** **01**	—		
Mean DBP	1.00	0.98–1.02	0.98	Not selected		
Mean heart rate	0.99	0.97–1.01	0.53	Not selected		
TTR-SBP	0.76	0.68–0.84	**<0** **.** **001**	0.78	0.67–0.90	0.001
TTR-DBP	0.59	0.28–1.25	0.17	Not selected		
TTR-heart rate	0.48	0.24–0.94	**0** **.** **03**	—		
NYHA class III/IV	1.20	0.60–2.39	0.60	—		
Stroke	2.32	1.14–4.71	**0** **.** **02**	3.28	1.49–7.21	0.003
Diabetes	0.37	0.80–2.34	0.24	Not selected		
Hyperlipidemia	1.17	0.53–2.59	0.70	Not selected		
Kidney disease	1.12	0.48–2.62	0.79	Not selected		
History of MI	1.08	0.53–2.21	0.83	Not selected		
Hyperuricemia	1.07	0.59–1.91	0.83	Not selected		
LVEF	1.01	0.99–1.04	0.27	Not selected		
Ast	1.01	0.99–1.02	0.34	Not selected		
Alt	1.00	0.99–1.01	0.49	Not selected		
Creatinine	1.00	0.99–1.01	0.60	Not selected		
LDL	0.80	0.55–1.18	0.26	Not selected		
HDL	0.87	0.47–1.62	0.66	Not selected		
TC	1.18	0.93–1.51	0.18	Not selected		
TG	1.24	0.97–1.58	0.08	—		
Procedure time	1.00	0.99–1.01	0.16	Not selected		
Length of hospital stay	1.14	1.03–1.26	**0** **.** **01**	—		
No. of lesions	1.19	0.97–1.47	0.10	Not selected		
Multiple arterial grafts	1.43	0.83–2.47	0.20	Not selected		
LIMA used as graft	0.81	0.11–5.89	0.84	Not selected		

Bold values indicate statistically significant differences (*P* < 0.05).CI, confidence interval; HR, hazard ratio; SBP, systolic blood pressure; SBP-TTR, systolic blood pressure time in target range; LVEF, left ventricular ejection fraction; Ast, aspartate aminotransferase; Alt, alanine aminotransferase; LDL, low-density lipoprotein; HDL, high-density lipoprotein; TC, total cholesterol; TG, triglycerides and LIMA, left internal mammary artery.

### Characteristics and medication adherence according to SBP-TTR group

All patients were categorized into three groups according to SBP-TTR levels: 0%–50% (*n* = 205), >50%–75% (*n* = 163), and >75%–100% (*n* = 160). Compared to those with a lower SBP-TTR, patients in the higher TTR group were characterized by younger age, lower baseline BP, and a superior rate of DBP goal attainment. Patients with higher number of lesions and prolonged postoperative hospital stay were more likely failed attaining the SBP goal (Table 3).

**Table 3:** Characteristics of participants by TTR of SBP groups.

Characteristics	TTR groups			*P*-value
	0–50%	>50%–75%	>75%–100%	
	(*n* = 205)	(*n* = 163)	(*n* = 160)	
Demographic and clinical characteristics				
Age in yr	70.6 ± 10.3	64.5 ± 11.7	61.4 ± 14.0	**<0** **.** **001**
Female, %	48 (23.4)	36 (22.1)	34 (21.3)	0.88
BMI, kg/m^2^	25.4 ± 4.4	26.0 ± 5.4	25.0 ± 4.4	0.17
Smoke, %	40 (19.5)	21 (12.9)	21 (13.1)	0.14
Drinking, %	20 (9.8)	15 (9.2)	8 (5)	0.21
Mean SBP, mmHg	137.5 ± 25.4	126.7 ± 24.9	119.6 ± 22.6	**<0** **.** **001**
Mean DBP, mmHg	77.5 ± 10.9	75.8 ± 10.6	73.9 ± 10.3	**0** **.** **006**
Mean heart rate, bpm	67.8 ± 10.9	69.8 ± 12.6	69.5 ± 12.1	0.22
TTR-DBP, %	65.1 ± 35.7	91.5 ± 19.6	91.1 ± 20.6	**<0** **.** **001**
TTR-heart rate, %	80.8 ± 30.7	82.3 ± 31.8	75.9 ± 35.6	0.18
NYHA class III/IV, %	30 (14.6)	23 (14.1)	19 (11.9)	0.62
Medical comorbidity, *n* (%)				
Stroke	17 (8.3)	15 (9.2)	11 (6.9)	0.74
Diabetes	73 (35.6)	50 (30.7)	61 (38.1)	0.09
Hyperlipidemia	23 (11.2)	15 (9.2)	13 (8.1)	0.40
Kidney disease	18 (8.8)	14 (8.6)	13 (8.1)	0.89
History of MI	32 (15.6)	22 (13.5)	17 (10.6)	0.24
Hyperuricemia	54 (26.3)	37 (22.7)	37 (23.1)	0.29
Laboratory measurements				
LVEF, %	58.9 ± 11.5	58.5 ± 11.7	58.2 ± 12.9	0.88
Ast, u/L	25.1 ± 19.5	22.8 ± 9.7	22.6 ± 10.3	0.22
Alt, u/L	27.0 ± 29.8	22.6 ± 15.2	26.4 ± 22.2	0.21
Creatinine, μmol/L	83.2 ± 44.2	82.1 ± 65.5	82.3 ± 33.2	0.98
LDL, mmol/L	2.3 ± 0.79	2.4 ± 0.80	2.2 ± 0.75	0.20
HDL, mmol/L	1.3 ± 0.41	1.3 ± 0.45	1.4 ± 0.45	0.29
TC, mmol/L	4.6 ± 1.1	4.4 ± 0.97	4.3 ± 0.95	**0** **.** **02**
TG, mmol/L	1.6 ± 0.70	1.5 ± 0.77	1.5 ± 1.0	0.93
Angiographic and procedural characteristics				
Procedure time, min	152.3 ± 37.8	150.6 ± 35.5	152.0 ± 38.4	0.90
Length of hospital stay, d	9.8 ± 2.6	9.3 ± 2.5	9.2 ± 2.5	**0** **.** **04**
No. of lesions	4.3 ± 1.2	4.0 ± 1.1	3.7 ± 1.2	**<0** **.** **001**
Multiple arterial grafts, %	57 (27.8)	34 (20.9)	42 (26.3)	0.29
LIMA used as graft, %	203 (99.0)	161 (98.8)	157 (98.1)	0.81

Values are mean ± SD or *n* (%).Bold values indicate statistically significant differences (*P* < 0.05).

BMI, body mass index; TTR, time in target range; SBP, systolic blood pressure; DBP, diastolic blood pressure; NYHA, New York Heart Association; MI, myocardial infarction; LVEF, left ventricular ejection fraction; Ast, aspartate aminotransferase; Alt, alanine aminotransferase; LDL, low-density lipoprotein; HDL, high-density lipoprotein; TC, total cholesterol; TG, triglycerides and LIMA, left internal mammary artery.

At 3-month post-discharge assessments, patients with higher SBP-TTR demonstrated superior medication adherence for ACEIs/ARBs (45.6% vs. 36.8% vs. 29.8%; *P* < 0.008) (Table 4). At the 12-month follow-up, the adherence of patients with higher SBP-TTR remained higher for *β* receptor blockers (55.6% vs. 49.1% vs. 40.5%; *P* < 0.015), ACEIs/ ARBs (29.4% vs. 19.6% vs. 16.6%; *P* < 0.010) and calcium channel blockers (14.4% vs. 9.2% vs. 4.9%; *P* = 0.007). Adherence to both clopidogrel/ticagrelor and aspirin showed a gradual decline over time, with no significant differences observed across all groups.

**Table 4:** Medication adherence and usage according to SBP-TTR groups.

Medication	Total	TTR group			*P*-value
		0–50%	>50%–75%	>75%–100%	
		(*n* = 205)	(*n* = 163)	(*n* = 160)	
*β* receptor blockers					
Baseline	84.7%	88.1%	82.2%	83.9%	0.31
3rd month	70.3%	59.4%	70.6%	78.5%	<0.001
12th month	57.0%	43.8%	49.1%	73.7%	<0.001
ACEI/ARB					
Baseline	53.2%	54.4%	47.2%	57.1%	0.16
3rd month	40.5%	31.9%	36.8%	50.2%	<0.001
12th month	27.7%	16.2%	19.6%	42.9%	<0.001
Calcium channel blockers					
Baseline	15.7%	12.5%	14.1%	19.5%	0.15
3rd month	12.3%	8.1%	10.4%	17.1%	0.02
12th month	8.9%	4.4%	7.4%	13.7%	0.006
Statins					
Baseline	88.8%	87.5%	87.7%	90.7%	0.53
3rd month	62.5%	27.5%	73.6%	81.0%	<0.001
12th month	50.4%	19.4%	53.4%	72.2%	<0.001
Clopidogrel/ticagrelor					
Baseline	51.1%	53.1%	55.2%	46.3%	0.20
3rd month	30.7%	22.5%	40.5%	29.3%	0.002
12th month	21.2%	15.0%	26.4%	22.0%	0.04
Diuretics					
Baseline	44.9%	48.1%	47.9%	40.0%	0.20
3rd month	24.4%	17.5%	33.1%	22.9%	0.004
12th month	15.0%	10.0%	19.0%	15.6%	0.07
Aspirin					
Baseline	99.1%	99.4%	99.4%	98.5%	0.75
3rd month	82.2%	80.0%	87.1%	80.0%	0.14
12th month	66.1%	66.9%	64.4%	66.8%	0.86

ACEIs, angiotensin-converting enzyme inhibitors; ARBs, angiotensin II receptor blockers; TTR, time in target range.

In the multivariable linear regression analysis, none of the baseline medication classes was independently associated with subsequent SBP-TTR. Baseline use of *β*-blockers (*β* = 0.045; 95% CI, −0.020 to 0.111; *P* = 0.177), ACEIs/ARBs (*β* = −0.044; 95% CI, −0.092 to 0.005; *P* = 0.078), calcium channel blockers (*β* = −0.008; 95% CI, −0.069 to 0.052; *P* = 0.791) and the other medications were not significantly associated with SBP-TTR. The complete results for all medication classes are provided in Supplementary Table S3. Additional adjustment for individual baseline medication classes did not materially alter the association between SBP-TTR and MACCEs. Across the medication-adjusted Cox regression models, the adjusted HRs ranged from 0.77 to 0.79 per 10% increase in SBP-TTR, and all associations remained statistically significant (Supplementary Table S4).

### Association of SBP-TTR and risk of cumulative clinical events

The cumulative incidence of events was significantly influenced by SBP-TTR, as shown in the Kaplan–Meier curves (Figure 2). Patients with the lowest TTR (0%–50%, red line) exhibited the highest cumulative incidence of events, reaching approximately 17% by the end of the study year. A higher SBP-TTR was associated with a significantly lower event probability over time (Log-rank *P* = 0.0014).

**Figure 2:**
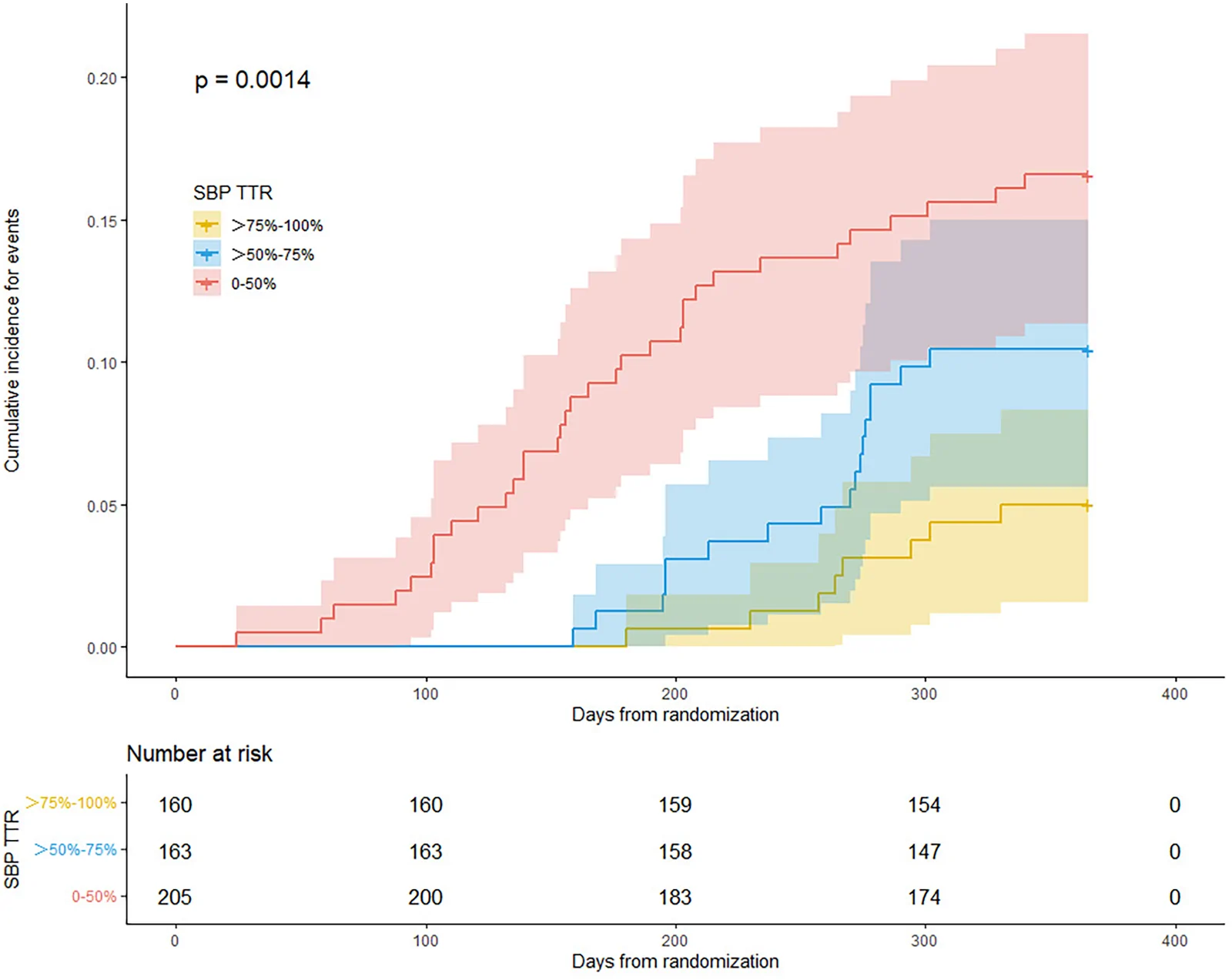
Kaplan–meier curves illustrating the incidence (95% confidence interval) of the composite of all-cause death, MI, stroke or repeat revascularization at 1 years under stratification according to SBP-TTR.

## Discussion

In this *post hoc* analysis of study in which 528 participants were digitally tracked for 12 months, we observed that inadequate BP control, especially SBP, is significantly associated with higher incidence of the composite of death, MI, stroke, or repeat revascularization at 1 year even after adjustment for age, sex, BMI, previous stroke, and mean SBP. Additionally, our study addressing the significance of enhancing medication adherence in the post-discharge management of patients with hypertension following coronary revascularization. These findings suggest that better BP control is essential for prognosis of patients with hypertension after CABG.

Hypertension is a pivotal comorbidity in coronary heart disease (CHD), with elevated BP linked to increased incidence of coronary events (16, 17). Both ACC/AHA and ESC/ESH guidelines therefore recommend stringent BP control for CHD patients to reduce cardiovascular risk (18, 19). A meta-analysis of 123 studies established that BP lowering significantly reduces vascular risk regardless of baseline BP or comorbidities, with each 10 mmHg SBP reduction lowering major cardiovascular event risk by 20% (20). Intensive control (SBP < 120 mmHg) further benefits high-cardiovascular-risk patients compared to standard target (<140 mmHg) (21). Substantial evidence confirms that post-revascularization, effective BP control—especially of SBP—is critical for long-term prognosis (22–24). A study on heart failure patients suggested an optimal SBP range of 120–130 mmHg in post-CABG patients (25). Consistently achieving guideline-directed medical therapy (GDMT) SBP targets is also associated with fewer cardiovascular events in CHD (26). Our findings reinforce that effective BP management is essential for optimizing clinical outcomes after CABG.

Population-based evidence indicates that BP variability rather than a single or average reading, is an independent prognostic factor for coronary heart disease, even after adjusting for mean BP (27, 28). *Post hoc* analyses suggested that the efficacy of antihypertensive treatment is contingent not only on the extent of BP reduction achieved but also on the stability of this effect over time, namely, the consistency of long-term on-treatment control (27, 29). Visit-to-visit BP variability demonstrated a significant association with both the progression of coronary atheroma and adverse clinical outcomes, with SBP variability being a particularly prominent factor (30). In patients undergoing CABG, day-to-day SBP variability was significantly associated with a heightened risk of both mortality and MACCEs, even after adjusting for mean BP and key clinical and demographic covariates (31). Proposed mechanism implicates increased oscillatory shear stress, a result of high BPV, initiating a sequence of events encompassing direct vascular endothelial injury and the progression of atherosclerosis, which collectively elevate the risks of stent thrombosis and impaired bridge vessel patency (32).

As a novel indicator of BP variability, TTR has been widely adopted in recent years as a key metric for evaluating clinical prognosis and stratifying risk in cardiovascular disease patients (15, 33, 34). Most studies calculated TTR primarily based on SBP, with considerable variation in the target ranges and monitoring durations. Despite the ongoing debate regarding the ideal SBP target, researches have consistently demonstrated that a greater TTR is strongly associated with a reduced risk of adverse cardiovascular events. Within an SBP target range of 110–130 mmHg, each SD increase in TTR corresponded to a respective 15% reduction in all-cause mortality and an 18% reduction in cardiovascular mortality (34). Evidence from previous meta-analysis indicates that elevated long-term BP variability independently predicts cardiovascular disease events (HR: 1.18, 95% CI: 1.07 to 1.30) and all-cause mortality (HR: 1.15, 95% CI: 1.09 to 1.22), with the magnitude of these associations exceeding that of mean BP (28). Our analysis revealed a graded association between TTR and risk. Relative to the reference group (TTR 75%–100%), the risk increased as TTR decreased, with the steepest gradient observed in the 0%–50% TTR group. This result aligns with prior research in underscoring the prognostic importance of BP control duration.

From a clinical perspective, SBP-TTR could be automatically calculated from longitudinal home BP measurements collected through digital post-discharge management platforms and displayed alongside conventional BP metrics. A persistently low or declining SBP-TTR may prompt clinicians to review BP measurement quality, medication use, treatment tolerability, and the need for therapeutic adjustment. SBP-TTR may also provide complementary prognostic information beyond conventional measures such as mean SBP and BP variability. Mean SBP primarily reflects the average BP level, whereas BP variability characterizes fluctuations between measurements. In contrast, SBP-TTR captures the proportion of time during which BP remains below a prespecified target threshold. Previous studies have shown that SBP-TTR remained associated with cardiovascular outcomes after adjustment for mean SBP or SBP variability, while the combined assessment of TTR and BP variability further improved risk stratification. However, because the present study did not formally compare the predictive performance of these BP metrics, further studies are needed to determine the incremental and comparative prognostic value of SBP-TTR in patients after CABG.

SBP-TTR was defined using the upper threshold applied in the digital management protocol and was intended primarily to reflect sustained avoidance of uncontrolled systolic hypertension. Previous studies have used both one-sided BP targets and bounded SBP ranges; therefore, no uniform lower boundary for SBP-TTR has been established (15, 35, 36). Nevertheless, our definition may have classified periods of potentially low SBP as being within target. Given the reported J-shaped relationship between achieved BP and cardiovascular outcomes in patients with coronary artery disease (37), future studies should evaluate bounded SBP ranges and jointly consider SBP, DBP, hypotensive symptoms, and graft-related outcomes.

Continued use of several cardiovascular medications during follow-up was more frequent among patients with higher SBP-TTR. This finding is consistent with previous evidence that poorer antihypertensive medication adherence is associated with inadequate BP control and greater cardiovascular risk (38, 39). However, the exploratory analyses did not identify statistically significant independent associations between baseline use of individual medication classes and subsequent SBP-TTR. Moreover, additional adjustment for each baseline medication class did not materially attenuate the association between SBP-TTR and MACCEs. These findings should not be interpreted as indicating that medical therapy is unimportant, as sustained secondary prevention therapy after CABG has been associated with improved long-term outcomes (40, 41). A single assessment of baseline medication class may not adequately capture medication dose, treatment indication, subsequent intensification or discontinuation, and persistence during follow-up. Accordingly, our results indicate only that differences in baseline medication class did not materially account for the observed association between SBP-TTR and MACCEs.

Several limitations should be considered when interpreting our results. First, the association between SBP-TTR and MACCEs should be interpreted cautiously because of the retrospective observational design. Patients with higher SBP-TTR differed in several clinical and follow-up characteristics, suggesting that SBP-TTR may reflect not only longitudinal BP control but also underlying disease severity, treatment tolerability, self-management ability, access to follow-up care, and engagement with the digital management platform. Although several measured covariates were included in the multivariable analyses, residual confounding from incompletely measured or unmeasured factors, including frailty, socioeconomic status, medication dose and treatment changes, medication-taking behavior, and quality of postoperative care, cannot be excluded. Accordingly, SBP-TTR should be interpreted as a prognostic marker associated with MACCEs rather than as a causal determinant of clinical outcomes. Second, although model complexity was restricted and the results remained consistent in a parsimonious sensitivity model, the number of MACCEs relative to the number of model parameters was limited. Some degree of model overfitting therefore cannot be excluded, and the multivariable estimates require confirmation in larger prospective cohorts. Third, medication use during follow-up was patient-reported rather than verified using objective measures and may have been affected by recall, reporting, and social desirability bias (42). Moreover, medication use and SBP-TTR were assessed during partly overlapping follow-up periods, precluding clear temporal ordering. Therefore, the observed relationships among medication use, SBP-TTR, and MACCEs should be interpreted as exploratory rather than as evidence of a causal or mediating pathway. Finally, this study included only patients with hypertension after CABG and excluded patients undergoing PCI, which may limit the generalizability of the findings (43). Prospective studies with more comprehensive covariate assessment, objective medication-use measures, and standardized monitoring protocols are needed to determine whether interventions designed to improve SBP-TTR can improve outcomes after CABG.

## Conclusions

Higher SBP-TTR is associated with improved clinical prognosis in hypertensive patients after CABG. While enhancing medication adherence represents a promising strategy to increase target achievement, its direct link to cardiovascular outcomes warrants further investigation in larger cohorts.

## Data Availability

The original contributions presented in the study are included in the article/Supplementary Material, further inquiries can be directed to the corresponding author/s.
