# Supplementary material for: Prognostic implication of time in target range of systolic blood pressure in patients with hypertension after coronary artery bypass grafting
Source: Front Cardiovasc Med. 2026 Sep 21;13:1869459. doi: 10.3389/fcvm.2026.1869459 (PMC13634840; doi:10.3389/fcvm.2026.1869459)
Supplement: Supplementary file 1 [file Table1.docx]

**Table S1** Completeness and Frequency of Blood Pressure Measurements During the 12-Month Follow-up

| **Measurement characteristic** |  | **Overall** |
| --- | --- | --- |
| Number of patients |  | 528 |
| Completed BP measurement sessions per patient, median (IQR) |  | 581.5 (518.5-642.0) |
| Missing scheduled BP measurement sessions, % |  | 20.2 |
| Valid measurement days per patient, median (IQR) |  | 298 (272-326) |
| Daily measurement frequency per patient, median (IQR), sessions/day |  | 1.6 (1.4-1.8) |
| Within-patient coefficient of variation in daily measurement frequency, median (IQR), % |  | 49.0 (36.0-61.2) |
| Longest consecutive period without a valid measurement day, median (IQR), days |  | 9 (7-12) |

Abbreviations: IQR, Interquartile Range.

**Table S2** Sensitivity analysis using alternative SBP thresholds

| **Analysis** | **SBP threshold, mmHg** | **Effect estimate** | **Adjusted HR** | **95% CI** | ***P* value** |
| --- | --- | --- | --- | --- | --- |
| Primary analysis | 140 | Per 10% increase in SBP-TTR | 0.78 | 0.67–0.90 | 0.001 |
| Sensitivity analysis | 130 | Per 10% increase in SBP-TTR | 0.73 | 0.60–0.89 | 0.002 |

Abbreviations: CI, confidence interval; HR, hazard ratio; SBP, systolic blood pressure; SBP-TTR, systolic blood pressure time in target range.

**Table S3** Associations of baseline medication use with subsequent SBP-TTR

| **Baseline medication** | **β coefficient** | **95% CI** | ***P* value** |
| --- | --- | --- | --- |
| β receptor blockers | 0.045 | -0.020-0.111 | 0.177 |
| ACEI/ARB | -0.044 | -0.092-0.005 | 0.078 |
| CCB | -0.008 | -0.069-0.052 | 0.791 |
| Statins | -0.028 | -0.104-0.049 | 0.478 |
| Clopidogrel/ticagrelor | 0.012 | -0.037-0.061 | 0.623 |
| Diuretics | 0.006 | -0.025-0.038 | 0.704 |
| Aspirin | -0.008 | -0.248-0.231 | 0.946 |

Abbreviations: ACEI, angiotensin-converting enzyme inhibitor; ARB, angiotensin receptor blocker; CCB, calcium channel blockers; CI, confidence interval; SBP-TTR, systolic blood pressure time in target range.

**Table S4** Association between SBP-TTR and MACCEs after additional adjustment for baseline medication use

| **Additional baseline medication included** | **Adjusted HR per 10% increase in SBP-TTR** | **95% CI** | ***P* value** |
| --- | --- | --- | --- |
| None (primary model) | 0.78 | 0.67-0.90 | 0.001 |
| β receptor blockers | 0.78 | 0.67-0.90 | 0.001 |
| ACEI/ARB | 0.78 | 0.67-0.90 | 0.001 |
| CCB | 0.79 | 0.67-0.92 | 0.002 |
| Statins | 0.77 | 0.66-0.90 | 0.001 |
| Clopidogrel/ticagrelor | 0.77 | 0.66-0.89 | <0.001 |
| Diuretics | 0.78 | 0.66-0.90 | 0.001 |
| Aspirin | 0.78 | 0.67-0.90 | 0.001 |

Abbreviations: ACEI, angiotensin-converting enzyme inhibitor; ARB, angiotensin receptor blocker; CCB, calcium channel blockers; CI, confidence interval; HR, hazard ratio; MACCEs, major adverse cardiovascular and cerebrovascular events; SBP-TTR, systolic blood pressure time in target range.

**Table S5** Sensitivity analysis using a parsimonious multivariable Cox regression model

| **Variable** | **Adjusted HR** | **95% CI** | **P value** |
| --- | --- | --- | --- |
| SBP-TTR, per 10% increase | 0.79 | 0.70-0.89 | <0.001 |
| Age, per year | 1.02 | 1.00-1.05 | 0.061 |
| Sex | 1.15 | 0.60-2.18 | 0.676 |
| BMI, per kg/m² | 1.05 | 1.02-1.09 | 0.006 |
| Mean SBP, per 1mmHg increase | 1.01 | 0.99-1.02 | 0.341 |
| Stroke | 2.43 | 1.19-4.97 | 0.015 |

Abbreviations: BMI, body mass index; CI, confidence interval; HR, hazard ratio; SBP, systolic blood pressure; SBP-TTR, systolic blood pressure time in target range.

**Supplementary Methods**

**Calculation of SBP Time in Target Range**

Valid daily SBP observations were arranged chronologically. Between two consecutive observations, SBP was assumed to change linearly over time. If both SBP values were below 140 mmHg, the entire interval was classified as time in target range. If both values were at or above 140 mmHg, none of the interval was classified as time in target range. If the two values were on opposite sides of the threshold, the threshold-crossing time was calculated by linear interpolation, and only the portion of the interval below 140 mmHg was counted. Interval-specific durations below the threshold were summed and divided by the total evaluable time between consecutive valid observations:

$$\mathrm{SBP}\text{-}\mathrm{TTR}=\frac{\sum\mathrm{time}\text{ }\mathrm{with}\text{ }\mathrm{SBP}<140\text{ }\mathrm{mmHg}}{\sum\mathrm{evaluable}\text{ }\mathrm{follow}\text{-}\mathrm{up}\text{ }\mathrm{time}}\times100\%$$

.
